# Maladaptive Rumination as a Transdiagnostic Mediator of Vulnerability and Outcome in Psychopathology

**DOI:** 10.3390/jcm8030314

**Published:** 2019-03-05

**Authors:** Maria Luca

**Affiliations:** Department of Medical, Surgical Sciences and Advanced Technologies “G.F. Ingrassia”, University of Catania, Via S. Sofia 78, 95125 Catania, Italy; lucmaria@tiscali.it

**Keywords:** rumination, psychopathology, emotion regulation, metacognition, psychosis, eating disorders, alcohol dependence

## Abstract

Background: Maladaptive rumination is a form of negative repetitive thinking which has attracted the interest of researchers, as it is considered a cognitive vulnerability to depression. Some of the original beliefs regarding rumination, in particular its exclusive link with depression, have been questioned in the light of research findings. At present, the very concept of rumination is still unclear, so research has been investigating this topic from different, and somewhat inconsistent, perspectives. Methods: A literature review was performed in order to outline some core characteristics of rumination, explain its determinants, and discuss its possible role as a transdiagnostic mediator of vulnerability and outcome in psychopathology. Results: Maladaptive rumination could be interpreted as a dysfunctional coping strategy strictly linked to emotion regulation and metacognition that may occur in several psychopathological conditions, such as psychosis, eating disorders, and alcohol dependence. Conclusion: Evidence allows the interpretation of maladaptive rumination as a transdiagnostic mediator of vulnerability and outcome in psychopathology. Therefore, investigating it from a dimensional perspective may represent a valid research strategy.

## 1. Introduction

Repetitive thoughts in response to life events or distressing feelings frequently occur in the general population and can represent either adaptive or maladaptive strategies [1]. Among the latter, rumination has attracted the interest of many researchers, as it is considered to be an important cognitive vulnerability for depression [2]. There are different theories of rumination, among which the most renowned is the response styles theory (RST) by Nolen-Hoeksema (1991), which considers ruminative thinking to be a response to negative affect: “ruminative responses to depression can be defined as behaviors and thoughts that focus one’s attention on one’s depressive symptoms and on the implications of these symptoms” [3] (p. 569). According to this theory, when individuals experience negative affect, they can engage in rumination and be “trapped” in a vortex of endless questions regarding the distressing condition (i.e., “why do I get depressed when other people don’t?”) or, on the contrary, they can put distraction into practice (i.e., focusing on neutral aspects, which in experimental settings include geographic locations), thus diverting the mind from current distress [3]. The RST states that rumination impairs problem-solving abilities and worsens the outcome of depression, while distraction relieves depressive symptoms and stimulates problem-solving [3]. In order to verify these assumptions, extensive research pertaining to the relationship between rumination, distraction, and depression followed. In support of the RST, it has been demonstrated that rumination represents a vulnerability factor for depression [4], determines longer and more severe depressive episodes [5,6], increases suicide risk [7], and impairs social problem-solving abilities [8]. In fact, being a form of maladaptive self-focus, rumination seems to favor the onset and persistence of depressive symptoms and could even mediate the predictive relationships between other risk factors (i.e., history of past depression, negative cognitive styles) and subsequent major depressive episodes [4,9]. In a prospective study, ruminative thinking was associated with the presence and duration of suicidal ideation [7]. In addition, rumination has been demonstrated to relate to the deficits in social problem-solving characterizing depression [8]. It is known that effective problem-solving requires a certain memory specificity, enabling the individual to recollect personally experienced past events. However, patients suffering from depression tend to recall over-general memories, hence referring to an extended period of time rather than to a precise moment [8,10]. Interestingly, rumination has been demonstrated to facilitate and maintain over-general memory [11], such that the consequential deficit in memory specificity could mediate the relationship between rumination and poor problem-solving [8]. On the other hand, ruminators facing a problem could consider it to be too difficult while showing no confidence in their abilities, thus displaying a reduced interest in solving it [11,12]. While the studies discussed here reported evidence that supports the RST, some aspects of the theory have been questioned. In particular, distraction has not been consistently demonstrated to impact depression outcome [13,14]. Moreover, the theory does not consider the importance of metacognitive beliefs (appraisals of rumination) as triggers of ruminative thinking [14]. Most importantly, while the RST considers rumination to be a response to depressive symptoms, evidence suggests that rumination is not exclusively linked to depression, crucially influencing vulnerability and outcome in several psychopathological conditions [15,16,17], as detailed later. Other theories of rumination define it as a response to perceived failure [18] or as a subset of worry within a larger model (self-regulatory executive function (S-REF) model) of emotional disorder including emotion regulation and metacognitive beliefs [19,20]. Also, regarding these theories, evidence suggests the need to refine some assumptions [14]. In particular, rumination seems to occur even in the absence of perceived failure [4]. Moreover, according to literature, rumination should be distinguished from worry, the latter being oriented towards the future and the former being mostly past-oriented due to its focus on self-relevant information [21,22]. As a matter of fact, the very concept of rumination is still unclear, so research has been investigating this topic from different, and somewhat inconsistent, perspectives. To further complicate matters, the supposed negative connotation of ruminative thinking described here has been recently questioned due to conflicting data regarding its effects on cognitive processing and problem solving. The inconsistencies could be due to the fact that rumination is probably more complex than previously believed, encompassing positive aspects as well as negative [23]. In fact, an event-related deliberate rumination seems to represent an adaptive way to ponder over severe stressors, allowing the individual to search for meanings and solutions, ultimately promoting post-traumatic growth [24]. In this context, some questions arise:

1. Regardless of the different theories, can a practical definition of rumination be outlined?
2. What is the role of metacognitive beliefs in the occurrence of rumination?
3. Could rumination be considered as a transdiagnostic mediator of vulnerability and outcome in psychopathology and, therefore, be investigated from a dimensional perspective?

**Figure 1:**
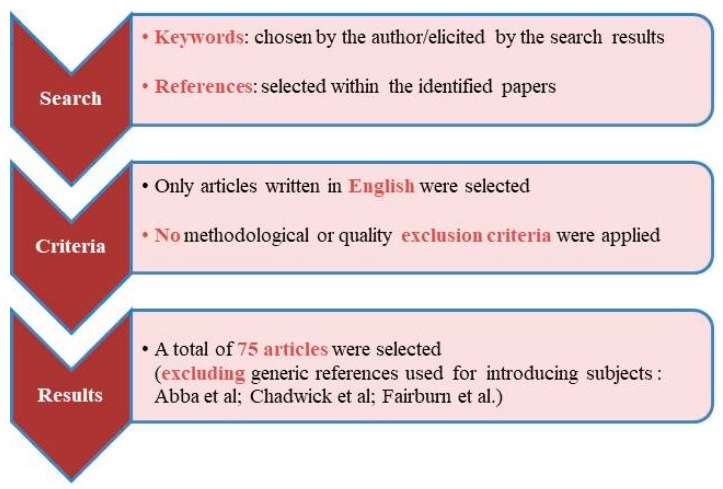
The figure shows how the bibliographic search was conducted.

In an attempt to answer these questions, a bibliographic search using the U.S. National Library of Medicine’s PubMed database and Google Scholar was performed (for more details, see Table 1 and Figure 1). Not presuming to address all the psychopathological conditions in one paper, I will only focus on the disorders in which the importance of rumination in terms of vulnerability and outcome has been repeatedly reported, namely psychosis [17], eating disorders [16], and alcohol dependence [15]. 

## 2. Towards a Practical Definition: Core Characteristics of Rumination

Consistent with the RST, one of the most-used measures of rumination is the Ruminative Responses Scale (RRS) [25], a 22-item self-report tool. While some items specifically investigate maladaptive (i.e., think “Why can’t I handle things better?”) and adaptive (i.e., analyze recent events to try to understand why you are depressed) thinking styles, other items have been criticized because of their overlap with measures of depressive symptoms, such as the Beck Depression Inventory (BDI) [14,26]. For example, the RRS item “Think about your feeling of fatigue and achiness” is reminiscent of how BDI addresses fatigue. In order to overcome these drawbacks, Treynor et al. (2003) performed a psychometric analysis on the measure of rumination unconfounded with depression content, thus obtaining a two-factor model of rumination, with brooding and self-reflection as two respectively maladaptive and adaptive components of ruminative thinking [27]. As a matter of fact, the paradox that self-focus could lead to both adaptive and maladaptive consequences has intrigued many researchers, and could be explained by this new vision of rumination, where maladaptive forms of self-focus could counteract the protective effects of the adaptive ones [28]. In particular, self-reflection allows the individual to analyze matters from a self-distanced perspective, focusing more on the causes than on the consequences, thus favoring insight and problem-solving [29,30]. On the contrary, brooding implies the passive comparison between current and desired status from a self-immersed perspective [27,29,30]. Being a form of self-focus, rumination could potentially be adopted in various depression-unrelated scenarios. In fact, it has been proven to occur in response to life events [9] and even in relation to positive emotions [31]. It is possible that the so-called “positive rumination” (ruminative thinking in response to positive emotions) and brooding belong to the same process of affect amplification. However, people with high trait positive affect could be more prone to engaging in protective forms of rumination due to their tendency to focus on the good aspects of events [31]. From what has been discussed here, regardless of the different theories, some core evidence-informed characteristics of rumination can be outlined:

1. Rumination seems to incorporate a maladaptive (brooding) and an adaptive (self-reflection) component [27];
2. It could be triggered by both internal (happiness, sadness) and external (life events) stimuli [9,14,31];
3. From a depression-unrelated perspective, rumination could be re-defined as “the process of thinking perseveratively about one’s feelings and problems rather than in terms of the specific content of thoughts” [13] (p. 400) being a “stable, negative, broadly constructed way of responding to discrepancies between current status and target status” [14] (p. 126).

In light of what has been reported here, “rumination” could become an umbrella term encompassing different “ruminations”: an adaptive (self-reflection/reflective pondering) and a maladaptive (brooding) one, having differential effects (positive versus negative) on problem solving and emotion regulation [23]. This review will mostly focus on the negative aspects of rumination. Hence, from now on the term “rumination” will be used to refer to the maladaptive component of ruminative thinking.

## 3. Rumination as an Emotion Regulation Strategy

The path leading to rumination in response to distressing conditions is certainly complex. However, it seems that people with a low sense of mastery over life events are more prone to brooding [32]. Consistent with these data, rumination can be interpreted as a dysfunctional emotion regulation strategy, leading to the defensive avoidance of emotion processing in response to life events or distressing feelings [9,33,34]. Unfortunately, avoidance coping not only impairs the ability to recover from stress, but it also exposes the individual to future stressors as in a vicious cycle [35]. More specifically, ruminators seem to be inclined to create stressful interpersonal environments, particularly if they constantly seek reassurance from those around them [36]. In fact, despite the possibility that sharing inner feelings with loved ones characterizes good-quality relationships, co-rumination entails high levels of distress for the people involved, to such an extent as to mediate the “contagion” of anxious-depressive symptoms, especially among youth [37,38]. As a matter of fact, the social nature of rumination itself could prompt the use of emotion-based coping strategies in response to social stressors, thus eliciting negative affect and anxiety [39]. I would like to remark that, generally speaking, rumination does not coincide with dysfunctional coping. This review mostly focuses on its maladaptive components, but it is worth remembering that a deliberate, not negatively focused, and voluntarily controlled rumination (self-reflection/reflective pondering) could represent an adaptive coping strategy favoring insight and problem solving, as well as enhancing self-awareness and self-regulation [24,29,30,40]. However, since pondering and brooding can stimulate each other, it is difficult to clearly state to what extent the adaptive mechanisms, being counteracted by brooding, become maladaptive [23,28,29]. Considering exclusively the maladaptive role of rumination, it seems reasonable to ask why people ruminate and, most importantly, why should they insist on using a maladaptive strategy—do they have any control over ruminative thinking? Metacognitive beliefs, discussed below, could shed light on this issue.

## 4. Metacognitive Beliefs as Triggers of Ruminative Thinking

The previously cited S-REF model consists of a multi-level architecture that considers rumination within a larger context of an emotional disorder strictly linked to self-knowledge [19,20,41]. More specifically, the model explains the emotion-related processing strategies through three interacting levels, namely, lower-level processing network, supervisory executive system, and self-knowledge. Lower-level processing accounts for the generation of automatic thoughts occurring in certain conditions, such as during a depressive episode. These thoughts activate the supervisory executive system with the consequential appraisal of the stimulus and the analysis of possible discrepancies between personal goals and the current situation. If discrepancies are detected, the search for coping strategies begins [41]. In the S-REF model, the key for the persistence of ruminative thinking lies in the executive control, which is largely driven by self-knowledge, the latter influencing metacognitive experiences [41], consisting in the “conscious cognitive or affective experiences that accompany and pertain to any intellectual enterprise” [42] (p. 906). More specifically, if a person believes that analyzing their thoughts and feelings is important (metacognitive self-knowledge), they will be more likely to engage in dysfunctional emotion-focused coping (rumination) at the expense of other, more adaptive, strategies [41]. However, if on the one hand ruminators could incorrectly interpret rumination as a way to “find out solutions”, on the other hand they adequately perceive its harmful consequences in terms of interpersonal relationships as well as its uncontrollability [43,44]. Dramatically, these negative beliefs regarding rumination determine feelings of hopelessness [43,44] that further stimulate ruminative thinking [41,43,44]. The reported data allow the interpretation of maladaptive rumination as a dysfunctional coping strategy that, by its very nature, could potentially occur in various psychopathological conditions, as detailed below.

## 5. The Transdiagnostic Nature of Rumination

Negative repetitive thinking, representing a maintaining factor of emotional disorders, accompanies nearly all psychiatric conditions. In fact, apart from the extensive literature regarding the impact of rumination on depression and anxiety, evidence suggests that ruminative thinking plays a role in a variety of psychopathologies [45]. However, rumination seems to have been repeatedly proven to be a mediator of vulnerability and outcome in psychosis [17], eating disorders [16], and alcohol dependence [15], since most of the articles obtained through the bibliographic search covered these conditions. Before deepening the discussion on these disorders, I would like to clarify the choice and meaning of referring to rumination as a “mediator”—psychiatric phenomena are extremely complex, and many variables could concur to their onset and maintenance. Hence, the term mediator aims to underline how rumination could influence vulnerability (as a risk factor) and outcome (as a maintaining factor) in psychopathology both directly (in itself) and indirectly (i.e., moderating the relationship between other cognitive vulnerabilities and symptoms).

### 5.1. Rumination and Psychosis

Coping with psychotic experiences (i.e., voices) encompasses mindfulness as a means to reach awareness of such experiences without being overwhelmed by them [46]. The stages leading to adequate coping are: awareness of psychosis; allowing psychotic experiences to come and go without reacting to them; empowerment through the acceptance of the self [46,47]. It is easy to envision that rumination interferes with these stages, leading to an unhealthy focus on the symptoms, their social consequences, and the negative emotions they provoke [48], thus favoring the occurrence of depressive symptoms and negative self-beliefs [48,49]. The resulting rumination-dependent impairment of the management of psychotic experiences could increase psychosis-related distress, even if literature offers conflicting results regarding this issue [17,50,51]. It is worth considering that patients with psychosis frequently experience feelings of shame that further stimulate ruminative thinking [52], the latter being considered to mediate the relationship between shame and auditory verbal hallucinations [53]. More specifically, ruminative thinking seems to determine an excessive attention towards shame-related events and enhance hallucination-proneness [53,54]. Moreover, rumination creates fertile ground for the maintenance of delusional beliefs, since it deprives the individual of the cognitive flexibility needed to disconfirm them [55,56]. Not only positive symptoms, but also negative ones (emotional withdrawal and stereotyped thinking in particular) have been demonstrated to relate to rumination [57]. These data support the role of rumination in the occurrence [17,54] and persistence [55,56] of psychotic experiences.

### 5.2. Rumination and Eating Disorders

Eating disorders are characterized by an over-evaluation of shape and weight [58]. Ruminative thinking is frequent among people suffering from eating disorders and focuses on disorder-related themes [59,60]. On the one hand, rumination could represent a vulnerability factor for both bulimia and anorexia, predicting the occurrence of eating-related symptoms, such as binging and starvation [60,61,62,63]. On the other hand, it seems to maintain dysfunctional eating habits, triggering binge eating and compensatory behaviors as a form of escapism from ruminative self-awareness [63]. In addition, rumination has been reported to enhance body dissatisfaction [64] and facilitate eating binges in response to distress [65]. Moreover, it has been found to relate to perfectionism [66] and low self-esteem [67], two important maintaining factors of eating disorders [58]. In summary, rumination could mediate vulnerability [62] and outcome [63] in eating disorders, as well.

### 5.3. Rumination and Alcohol Dependence

Alcohol-dependent individuals seem to be prone to abstract thinking, which contributes to their interpersonal deficits [68,69]. The latter are precipitated by maladaptive self-beliefs, such as excessively high standards for social performance and negative self-appraisals, which maintain alcohol consumption and facilitate the occurrence of co-morbid social anxiety and depression [70]. Unsurprisingly, ruminative thinking has been repeatedly demonstrated to favor and maintain maladaptive self-beliefs that, in turn, relate to higher levels of rumination preceding and following events of interpersonal exchange [71,72,73]. From a clinical point of view, ruminators are at high risk of being prospectively classified as problem drinkers [74]. In fact, rumination seems to mediate the vulnerability to alcohol dependence, influencing drinking status and level of alcohol consumption [15], enhancing weekly alcohol use [75] and prompting alcohol consumption as a dysfunctional way to deal with stress [76]. In addition, ruminative thinking could worsen the outcome of alcohol dependence, exacerbating craving [77] and increasing the risk of aggressive behaviors under the effects of alcohol, most probably through the reduction of the ability to distract from anger-provoking stimuli [78].

As previously stated, for the sake of brevity, psychosis, eating disorders, and alcohol dependence were chosen as examples of psychopathology because most of the articles obtained through the bibliographic search pertained to these conditions. However, it would be a mistake to consider the impact of rumination as limited to these disorders. In fact, maladaptive rumination (negative repetitive thinking) plays a role in a variety of psychopathological conditions, such as post-traumatic stress disorder, social phobia, obsessive-compulsive disorder, hypochondriasis, and bipolar disorder. The specific focus of the ruminative thinking may vary being linked to the disorder-related concerns (i.e., trauma in post-traumatic stress disorder, performance in social interactions in social phobia), but its characteristics of repetitiveness, uncontrollability, and negative content remain unaltered, allowing the interpretation of rumination as a transdiagnostic process [45]. Interestingly, the impact of such a dimensional perspective of rumination would not be limited to the theoretical level, as detailed below.

## 6. Conclusions

Despite its limitations (in particular the lack of methodological and quality exclusion criteria for the bibliographic search as well as the free choice of references deemed to be relevant), this review tried to contribute to the current knowledge regarding rumination. The information reported in this paper could be summarized by simply stating that rumination has been gaining the dignity of a complex cognitive phenomenon whose importance could go beyond the diagnostic barriers. Hence, rumination should probably be “freed” from its tight bond with depression in order to be properly investigated. In fact, scholars studying rumination, regardless of the diagnostic boundaries, would have access to larger samples of ruminators and could verify the transdiagnostic efficacy of the psychological interventions. In conclusion, by virtue of these practical implications, addressing rumination from a dimensional perspective could represent a valid research strategy allowing current knowledge regarding this fascinating construct to increase.

## Figures and Tables

**Table 1:** Bibliographic search keywords.

Keywords
Adaptive self-reflection; maladaptive brooding
Protective effects of rumination
Rumination/ruminative thinking/negative repetitive thinking review
Rumination/ruminative thinking/negative repetitive thinking transdiagnostic factor
Rumination/ruminative thinking/negative repetitive thinking vulnerability factor
Self-reflection versus self-rumination
Rumination/ruminative thinking/negative repetitive thinking “AND” alcohol abuse; alcohol dependence
Anorexia; bulimia; binge eating; eating disorders
Avoidance coping; coping
Delusions; hallucinations; paranoid ideation; psychotic symptoms
Interpersonal stress
Life stressors
Problem solving
Psychiatric disorders
Psychiatric symptoms
Psychopathology
Self-beliefs
Social anxiety
